# Correlation between Antibodies to Bacterial Lipopolysaccharides and Barrier Proteins in Sera Positive for ASCA and ANCA

**DOI:** 10.3390/ijms21041381

**Published:** 2020-02-18

**Authors:** Aristo Vojdani, Elroy Vojdani, Martha Herbert, Datis Kharrazian

**Affiliations:** 1 Immunosciences Lab, Inc. 822 S. Robertson Blvd, Ste 312, Los Angeles, CA 90035, USA; 2 Department of Preventive Medicine, Loma Linda University, Loma Linda, CA 92350, USA; Datis_kharrazian@hms.harvard.edu; 3 Regenera Medical, 11860 Wilshire Blvd., Ste. 301, Los Angeles, CA 90025, USA; evojdani@gmail.com; 4 Martha Herbert, Pediatric Neurology, Massachusetts General Hospital, Rm CNY149-2nd Floor, Boston, MA 02114, USA; martha.herbert@mgh.harvard.edu; 5 Department of Neurology, Harvard Medical, Boston, MA 02115, USA; 6 Department of Neurology, Massachusetts General Hospital, Boston, MA 02129, USA

**Keywords:** IBD, lipopolysaccharide, zonulin+occludin, aquaporin, S100B, BBB permeability

## Abstract

Individuals with intestinal barrier dysfunction are more prone to autoimmunity. Lipopolysaccharides (LPS) from gut bacteria have been shown to play a role in systemic inflammation, leading to the opening of the gut and blood-brain barrier (BBB). This study aims to measure antibodies against LPS and barrier proteins in samples positive for anti-*Saccharomyces cerevisiae* antibodies (ASCA) and anti-neutrophil cytoplasmic antibodies (ANCA) and compare them with these same antibodies in controls to determine whether a correlation between LPS and barrier proteins could be found. We obtained 94 ASCA- and 94 ANCA-positive blood samples, as well as 188 blood samples from healthy controls. Samples were assessed for antibodies to LPS, zonulin+occludin, S100B, and aquaporin-4 (AQP4). Results show significant elevation in antibodies in about 30% of ASCA- and ANCA-positive sera and demonstrate positive linear relationships between these antibodies. The findings suggest that individuals positive for ASCA and ANCA have increased odds of developing intestinal and BBB permeability compared to healthy subjects. The levels of LPS antibodies in both ASCA- and ANCA-positive and negative specimens showed from low and moderate to high correlation with antibodies to barrier proteins. This study shows that LPS, by damaging the gut and BBBs, contribute to the extra-intestinal manifestation of IBD. We conclude that IBD patients should be screened for LPS antibodies in an effort to detect or prevent possible barrier damage at the earliest stage possible to abrogate disease symptoms in IBS and associated disorders.

## 1. Introduction

Inflammatory bowel disease (IBD) is a heterogeneous group of chronic inflammatory disorders of the gastrointestinal (GI) tract that has two main distinguishable forms, Crohn’s disease (CD) and ulcerative colitis (UC) [1]. According to the Centers for Disease Control and Prevention, CD can affect any part of the GI tract from the mouth to the anus, but it most often affects the portion of the small intestine before the large intestine/colon; UC, on the other hand, occurs in the large intestine and colon [2]. Another way to differentiate between CD and UC is that anti-*Saccharomyces cerevisiae* antibodies (ASCA) are associated with and used as biomarkers for CD, while anti-neutrophil cytoplasm antibodies (ANCA) are recognized as markers for UC [3]. 

*Saccharomyces cerevisiae*, also known as baker’s or brewer’s yeast, is the most commonly detected fungi in human fecal samples and likely originates from food [4]. ASCA are antibodies against antigens presented by the cell wall of the yeast *S. cerevisiae;* they are widely recognized as test markers for CD. For instance, in 2016 Huang et al. did a five-year follow-up study on 51 patients with CD compared to a control group. ASCA IgG had a sensitivity of 45.1%, a specificity of 100%, and a positive predictive value (PPV) of 100%, while ASCA IgA’s respective results were 35.3%, 91%, and 90% [5]. This is only one of many studies showing the clinical value and usefulness of ASCA as a predictive tool in the diagnosis of CD.

Anti-neutrophil cytoplasmic antibodies (ANCA) are a group of autoantibodies against antigens in the cytoplasm of neutrophil granulocytes and monocytes. ANCA are divided into cytoplasmic ANCA (c-ANCA), perinuclear ANCA (p-ANCA), and atypical ANCA (x- or a-ANCA) [6]. c-ANCA is detected in 80%–90% of patients with granulomatosis with polyangiitis and in some patients with glomerulonephritis, eosinophilic granulomatosis, cystic fibrosis, inflammatory bowel disease, rheumatoid arthritis, and other disorders. Proteinase is a specific antigen for c-ANCA. p-ANCA antigens include myeloperoxidase and a bacterial permeability increasing factor. p-ANCA is elevated in patients with IBD, rheumatoid arthritis, drug-induced vasculitis, autoimmune liver disease, and more [7]. ANCA are particularly associated with a heterogeneous group of conditions called ANCA-associated vasculitides or AAV [8]. a-ANCA is found in patients with other conditions, such as drug-induced AAV [9].

IBD has been associated with a greater risk for increased permeability of the intestinal barriers [10,11]. This increased permeability has itself been recognized as being involved in many disorders, particularly autoimmune diseases, such as type 1 diabetes, multiple sclerosis, and rheumatoid arthritis [12]. Increased intestinal, antigenic permeability has been found to play a key role in the development of various inflammatory and autoimmune disorders [13]. Menard et al. found that increased permeability to large molecules can have a deleterious effect by exacerbating inappropriate immune responses [14]. Klatt et al. showed that prior to developing the simian equivalent of human immunodeficiency virus (HIV), pigtail macaque monkeys had microbial translocations that were followed by significant damage to the structural barriers of their gastrointestinal tracts [15]. Increased gut permeability, or leaky gut, also enables enteric bacterial lipopolysaccharide (LPS) to penetrate into the bloodstream. The LPS by itself is already inductive of inflammation, but it also has the added detriment of triggering the production of cytokines, such as tumor necrosis factor-alpha, that have been shown to play a role in multiple autoimmunities [16]. This is one possible explanation for the increase of comorbidities seen in ulcerative colitis (UC) and Crohn’s disease (CD) [17,18,19,20,21,22]. 

Comorbidities of a neurological nature are not reported as frequently in conjunction with IBD [23], but any neurological dysfunction is a grave matter. Consequently, it is imperative to investigate a possible link between the translocation of LPS, as occurs in IBD, to a breakdown of the blood-brain barrier (BBB), which is often seen as a precursor of neuroautoimmunity [24]. 

### 1.1. Gut-Derived Lipopolysaccharides

LPS is a component of the surface membrane of Gram-negative bacteria found in the gastrointestinal tract. Gram-negative bacteria include *Escherichia coli*, *Salmonella*, *Shigella*, *Pseudomonas*, *Helicobacter*, *Legionella*, and *Wolbachia*. As an endotoxin, LPS increases the negative charge of the bacterial membrane and promotes the upregulation of pro-inflammatory cytokines, which contributes to gut dysbiosis [13,25,26,27,28,29,30]. Heavy colonization of Gram-negative bacteria is followed by the release of LPS, which infiltrates through the intestinal barrier into the systemic circulation [13]. Evidence indicates that bacterial LPS can elicit antibodies that cross-react with host nuclear materials [19]. Systemic LPS and the immune response against it has been shown to play a role in multiple disorders, including the opening of intestinal tight junctions, dysregulation of the BBB, neuroinflammation and neuroautoimmune disorders [25,31,32,33,34,35,36,37,38,39]. Candido et al. found that increased LPS translocation is one of many factors that can lead to dysbiosis [40]. Chelakkot et al. showed that mice fed a high-fat diet showed a three- to fourfold increase in serum LPS (metabolic endotoxemia), and that LPS itself is known to impact the increase in gut permeability [41].

### 1.2. Intestinal and Blood-Brain Barriers

The single-cell epithelial lining in the intestines forms the single, largest interface between the human body and the external environment with a surface area of 300-m^2^ (about the size of a tennis court) [42]. Comprised of epithelial cells connected by tight junction tissues (occludin, zonulae, claudin, etc.) this barrier plays an important role in maintaining health. The function of intestinal mucosa is far more than just the transportation of nutrients; it balances the needs for a barrier against a hostile environment factors. The intestinal mucosal cells absorb nutrients, regulate ion and water movements, and limit host contact with the massive intraluminal load of dietary antigens and microbial toxins [14]. An intact intestinal barrier is, therefore, critical to normal physiological function and the prevention of disease [43].

The BBB is a physical barrier between the brain and the circulating blood, formed by the arrangement of endothelial cells and tight junctions that line the capillaries supplying blood to the brain. It is a highly selective barrier. The BBB naturally permits the passage of essential metabolites, oxygen, carbon-dioxide, hormones, cytokines, and other messengers. However, it restricts the movement of all soluble materials greater than 400 Da from the blood across to the brain. Acting like a filter, the BBB excludes many potentially toxic compounds, such as xenobiotics (bacterial toxins, chemicals, mycotoxins), viruses, food antigens and peptides present in the circulation [44]. Despite the design of the BBB, systemic LPS enhances the entry of immune cells and even microorganisms across the intact BBB [45,46].

Astrocytes are characteristic star-shaped glial cells in the brain and spinal cord that protect the BBB. These cells secrete S100B, and their endfeet, which enwrap the BBB, abundantly express aquaporin-4 (AQP4). If the astrocyte is injured, S100B and AQP4 may be found in the bloodstream [47,48]. Once entering the bloodstream, these unique tissue proteins become the target for immune response and the production of antibodies against them.

Because intact barriers support and maintain the health of the individual, a breach of one of these essential body barriers is an invitation for autoimmune reactivity to self-tissue, including the brain. Banks and colleagues [49] pinpointed the neurological areas that became targets for inflammation after LPS had disrupted the BBB; these include the frontal cortex, thalamus, pons-medulla, and cerebellum. Recognized as the great inducer of neuroinflammation, LPS injections have been used in multiple animal models of neurological disorders from autism spectrum disorders to multiple sclerosis [32,33,37,50,51,52,53,54,55]. Furthermore, LPS antibodies have been found in the sera of patients diagnosed with Alzheimer’s disease, chronic fatigue, major depression, and schizophrenia [56,57,58,59,60]. Individuals with IBD have been shown to have increased blood levels of LPS [61,62,63]; therefore, they would be at greater risk for a breach of the BBB and neuroautoimmunity.

Thus, the aim of this study is to compare antibodies against LPS and barrier proteins in ASCA- and ANCA-positive samples versus controls and to investigate the correlation between them.

## 2. Results

A total of 376 sera were assessed for levels of IgG, IgM, and IgA isotype antibodies against LPS, zonulin+occludin, the BBB protein S100B, and the water channel protein AQP4.

**Figure 1:**
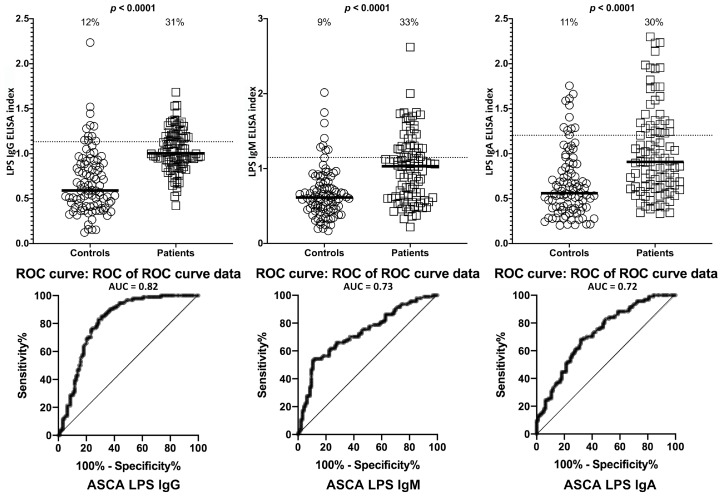
Percentage of elevation of IgG, IgM, and IgA antibodies against lipopolysaccharides (LPS) in 94 patients positive for ASCA and in 94 healthy controls. Dotted lines indicate the cutoff for positivity used in each assay, as calculated by ROC analysis. The percentage of elevation is indicated on top of each distribution, while bars indicate the corresponding median. The AUC for LPS IgG was 0.82, 0.73 for LPS IgM, and 0.72 for LPS IgA.

Data analysis was conducted with three separate groups that included ASCA-positive subjects (*n* = 94), ANCA-positive subjects (*n* = 94), and healthy control subjects (*n* = 188). Each group had separate measurements of the three isotype antibodies. Based on ROC analysis, the percentages of samples with significant elevation in antibody levels were evaluated. Compared to healthy controls or ASCA-negative specimens, the ASCA-positive sera showed significant elevation in IgG, IgM and IgA antibodies against LPS. While the % elevation of LPS antibodies in controls was between 9% (8/94) and 12% (11/94), ASCA-positive samples showed significant levels of LPS antibodies in 30% (28/94) to 33% (31/94) (Figure 1). *p*-values for all three isotype antibodies were <0.0001.

**Figure 2:**
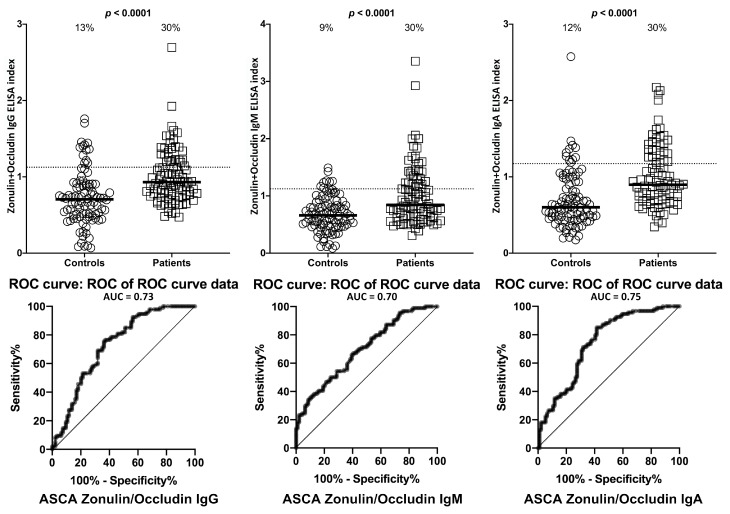
Percentage of elevation of IgG, IgM, and IgA antibodies against zonulin+occludin in 94 patients positive for ASCA and in 94 healthy controls. Dotted lines indicate the cutoff for positivity used in each assay, as calculated by ROC analysis. The percentage of elevation is indicated on top of each distribution, while bars indicate the corresponding median. The AUC for zonulin+occludin IgG was 0.73, 0.70 for zonulin+occludin IgM, and 0.74 for zonulin+occludin IgA.

The % elevation in IgG, IgM, and IgA antibodies against zonulin+occludin in the same 94 controls and 94 patients positive for ASCA at the cutoff for positivity used for each assay is shown in Figure 2. These % elevations for zonulin+occludin antibodies were between 9% (8/94) and 13% (12/94) in controls and 30% (28/94) in the ASCA-positive individuals with *p* < 0.0001 (Figure 2).

**Figure 3:**
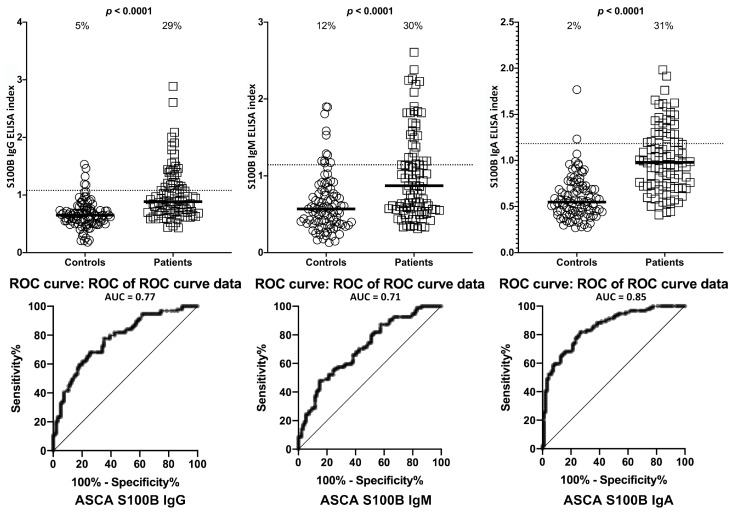
Percentage of elevation of IgG, IgM, and IgA antibodies against S100B in 94 patients positive for ASCA and in 94 healthy controls. Dotted lines indicate the cutoff for positivity used in each assay, as calculated by ROC analysis. The percentage of elevation is indicated on top of each distribution, while bars indicate the corresponding median. The AUC for S100B IgG was 0.77, 0.71 for S100B IgM, and 0.85 for S100B IgA.

Using the same criteria for the calculation of % of antibody elevation, while 29% (27/94) to 31% (29/94) of ASCA-positive samples showed significant elevations in these antibodies, 5% (5/94), 12% (11/94), and 2% (2/94) of controls had elevations in IgG, IgM, and IgA antibodies against S100B, with *p*-values < 0.0001 for all three determinations (Figure 3).

**Figure 4:**
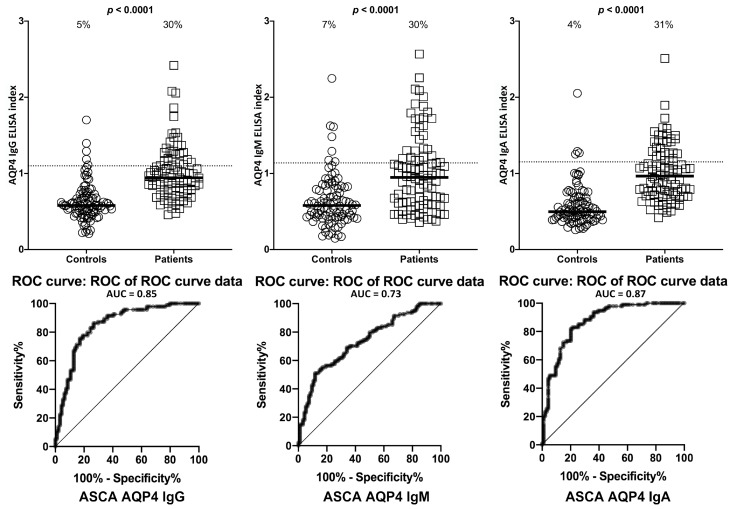
Percentage of elevation of IgG, IgM, and IgA antibodies against aquaporin-4 (AQP4) in 94 patients positive for ASCA and in 94 healthy controls. Dotted lines indicate the cutoff for positivity used in each assay, as calculated by ROC analysis. The percentage of elevation is indicated on top of each distribution, while bars indicate the corresponding median. The AUC for AQP4 IgG was 0.85, 0.73 for AQP4 IgM, and 0.87 for AQP4 IgA.

Similar results with minor variations were obtained when % elevation of antibodies was determined for AQP4, with *p* < 0.0001 (Figure 4).

Overall, there were statistically significant differences in mean antibody levels for LPS, zonulin+occludin, S100B and in AQP4 in ASCA-positive subjects compared to healthy controls for IgG, IgM, and IgA antibodies (*p* < 0.0001). The mean differences are illustrated in Figure 1, Figure 2, Figure 3 and Figure 4 by the black bars.

**Figure 5:**
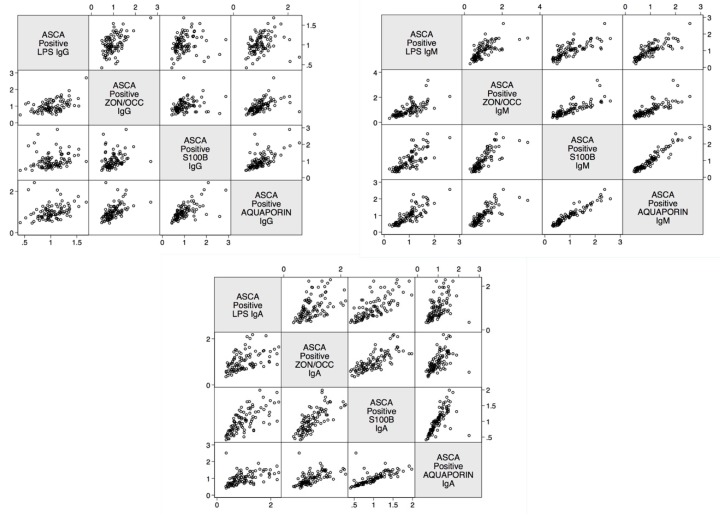
Scatter matrix plots illustrating positive linear relationships between barrier proteins for ASCA-positive subjects for IgG, IgM, and IgA. All linear relationships were statistically significant (*p* < 0.0001). ASCA—Anti-*Saccharomyces cerevisiae* antibodies; LPS—Lipopolysaccharides; ZON/OCC—Zonulin+Occludin; S100B—S100 calcium-binding protein B.

The linear relationship between LPS antibody and zonulin+occludin, S100B, and AQP4 was determined. The data presented in Figure 5 shows the positive linear relationship between these four determinations in ASCA-positive samples.

The odds ratio for developing barrier protein antibodies with ASCA positivity was also calculated. The odds for developing blood-brain barrier permeability (S100B and AQP4 antibodies) and intestinal barrier permeability (zonulin+occludin and LPS antibodies) were significantly higher for ASCA-positive subjects than the odds for healthy control subjects (Table 1). The highest odds ratio in ASCA-positive subjects was observed in AQP4 IgA, followed in descending order by LPS IgA, AQP4 IgG, S100B IgG, S100B IgA, zonulin+occludin IgA, and zonulin+occludin IgG. The lowest odds ratio for all four determinations was with the IgM isotype antibody (5–9), with the exception of LPS IgM (11).

The correlation coefficients between antibodies to LPS and barrier proteins and between gut and BBB proteins in ASCA-positive subjects were calculated, with the highest being between AQP4 IgM and S100B IgM (r 0.9), followed by zonulin+occludin IgM and AQP4 IgM (r 0.8), LPS IgM and S100B IgM (r 0.8) and zonulin+occludin IgA with S100B IgA (r 0.8) (Table 2). This correlation coefficient with a *p*-value of 0.2 was the least significant for LPS IgG and S100B IgG (r 0.2) and for LPS IgG and AQP4 IgG (r 0.3).

Similar results with minor variations were obtained when % elevation of antibodies was determined for AQP4, with *p* < 0.0001 (Figure 4).

**Figure 6:**
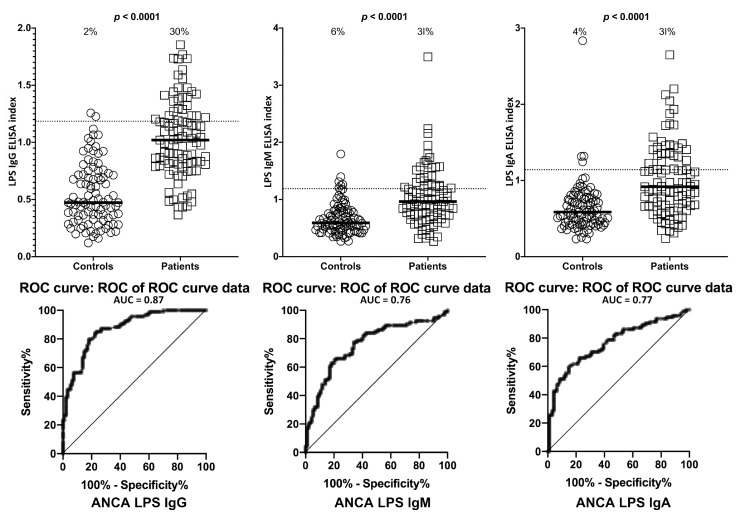
Percentage of elevation of IgG, IgM, and IgA antibodies against lipopolysaccharides (LPS) in 94 patients positive for ANCA and in 94 healthy controls. Dotted lines indicate the cutoff for positivity used in each assay, as calculated by ROC analysis. The percentage of elevation is indicated on top of each distribution, while bars indicate the corresponding median. The AUC for LPS IgG was 0.87, 0.76 for LPS IgM, and 0.77 for LPS IgA.

**Figure 7:**
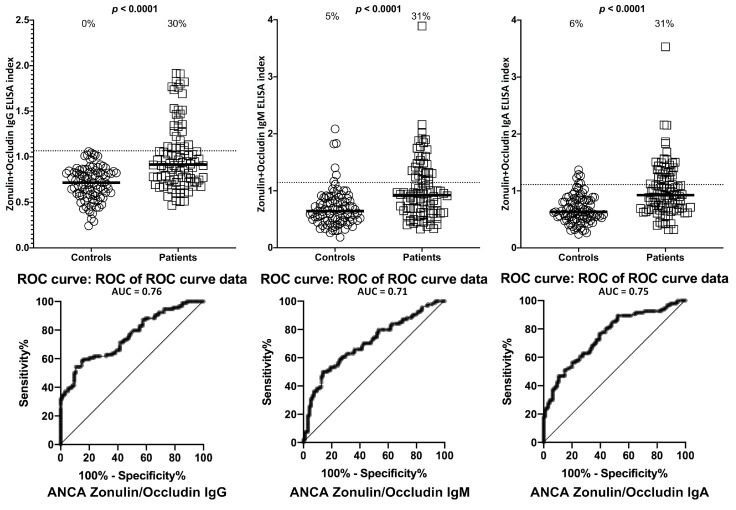
Percentage of elevation of IgG, IgM, and IgA antibodies against zonulin+occludin in 94 patients positive for ANCA and in 94 healthy controls. Dotted lines indicate the cutoff for positivity used in each assay, as calculated by ROC analysis. The percentage of elevation is indicated on top of each distribution, while bars indicate the corresponding median. The AUC for zonulin+occludin IgG was 0.76, 0.71 for zonulin+occludin IgM, and 0.75 for zonulin+occludin IgA.

**Figure 8:**
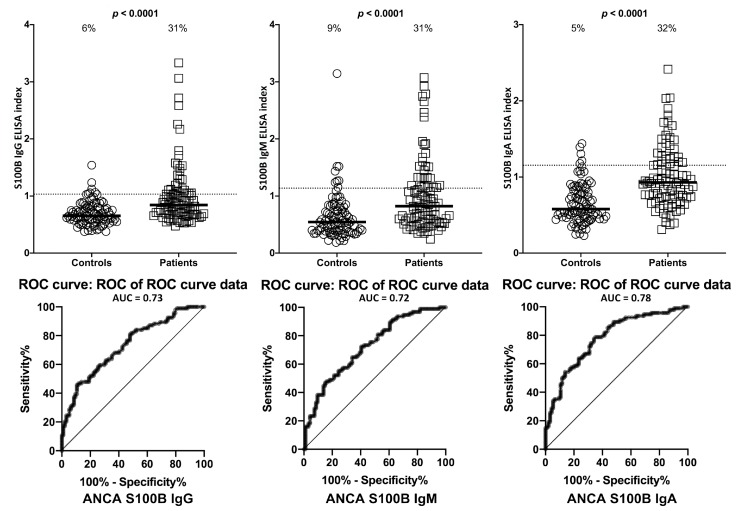
Percentage of elevation of IgG, IgM, and IgA antibodies against S100B in 94 patients positive for ANCA and in 94 healthy controls. Dotted lines indicate the cutoff for positivity used in each assay, as calculated by ROC analysis. The percentage of elevation is indicated on top of each distribution, while bars indicate the corresponding median. The AUC for S100B IgG was 0.73, 0.72 for S100B IgM, and 0.78 for S100B IgA.

**Figure 9:**
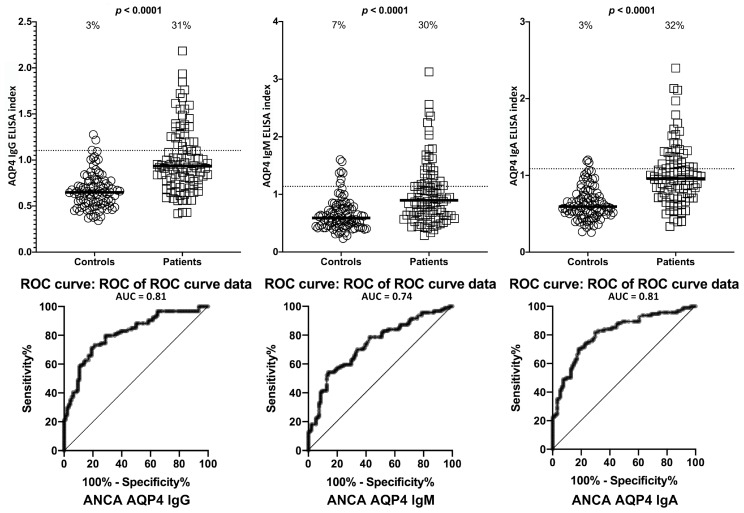
Percentage of elevation of IgG, IgM, and IgA antibodies against aquaporin-4 (AQP4) in 94 patients positive for ANCA and in 94 healthy controls. Dotted lines indicate the cutoff for positivity used in each assay, as calculated by ROC analysis. The percentage of elevation is indicated on top of each distribution, while bars indicate the corresponding median. The AUC for AQP4 IgG was 0.81, 0.74 for AQP4 IgM, and 0.81 for AQP4 IgA.

Setting the specificity at 31%, we found between 0%–9% of controls or ANCA-negative sera showed elevations in IgG, IgM, and IgA antibodies against LPS, zonulin+occludin, S100B and AQP4. This % elevation for all antibodies against all four antigens in ANCA-positive samples was 29%–33% with *p*-values < 0.0001 for all determinations (Figure 6, Figure 7, Figure 8 and Figure 9). The elevation percentages in IgG, IgM, and IgA antibodies for all four antigens and the actual numbers (*n*/94) for each are shown in Table 3.

**Figure 10:**
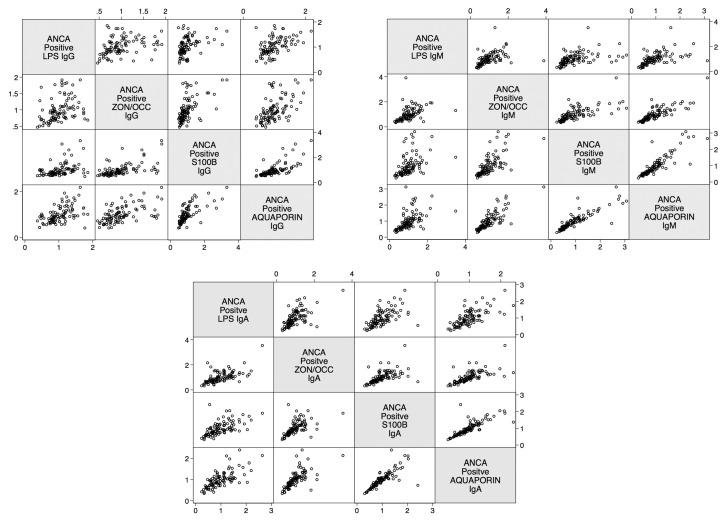
Scatter matrix plots illustrating positive linear relationships between barrier proteins for ANCA-positive subjects for IgG, IgM, and IgA. All linear relationships were statistically significant (*p* < 0.0001). ANCA—Anti-neutrophil cytoplasmic antibodies; LPS—Lipopolysaccharides; ZON/OCC—Zonulin+Occludin; S100B—S100 calcium-binding protein B.

The linear relationship between LPS antibody and gut and BBB proteins in relation to ANCA positivity was calculated. The data presented in Figure 10 shows the positive linear relationship between these four determinations in ANCA-positive samples.

The odds ratio for developing barrier protein antibodies with ANCA positivity was also calculated. The odds for developing blood-brain barrier permeability (S100B and AQP4 antibodies) and intestinal barrier permeability (zonulin+occludin and LPS antibodies) were significantly higher for ANCA-positive subjects than the odds for healthy control subjects (Table 4). Similar to the ASCA-positive subjects, the odds ratio for ANCA-positive subjects was most significant with AQP4 antibodies. This was followed in descending order by S100B IgA, LPS IgG, S100B IgG, zonulin+occludin IgG, LPS IgM, and zonulin+occludin IgA.

The correlation coefficients between antibodies to LPS, zonulin+occludin, S100B, and AQP4 in ANCA-positive samples was calculated. There were statistically significant correlations (*p*-values < 0.0001) and moderate-to-high r-values with intestinal barrier permeability and blood-brain barrier permeability for ANCA-positive subjects (Table 5). The highest correlation found was between AQP4 IgM and S100B IgM (r 0.9), followed by AQP4 IgA and S100B IgA (r 0.8), and zonulin+occludin IgM with AQP4 IgM (r 0.8). The lowest correlation found was between LPS IgG, IgM and zonulin+occludin IgG, IgM (r 0.3, r0.4), and LPS IgG, IgM with S100B IgG, IgM (r 0.4). *p*-values for all these determinations were < 0.0001 (Table 4).

We also screened 24 specimens from patients with celiac disease. We found that 9 out of these 24 sera were positive for ASCA IgA, and 4 out of the same 24 specimens were positive for ANCA IgA. This indicates that a subgroup of celiac disease patients may suffer from IBD, and vice versa.

## 3. Discussion

The aim of our study was to measure antibodies against LPS and barrier proteins in ASCA- and ANCA-positive samples and compare them with these same antibodies in controls to determine whether a correlation between LPS and barrier proteins could be found.

Our study found statistically significant differences in mean antibody levels in healthy controls compared to both ASCA- and ANCA-positive subjects for antibodies to both intestinal barrier and BBB proteins (Table 3). We also identified significant odds for both BBB and intestinal permeability for ASCA- and ANCA-positive subjects compared to the odds of healthy subjects. 

Because gut-derived LPS regulates intestinal tight junction permeability and plays an essential role in the induction of intestinal and systemic inflammatory disorders [60], we also measured the level of LPS antibodies in both ASCA- and ANCA-positive and negative specimens. Depending on the isotype antibody, our results showed from low and moderate to high correlation between LPS antibodies and barrier protein antibodies (Table 2 and Table 4). These antibodies were significantly higher in about one-third of ASCA- and ANCA-positive sera. Our results indicate that LPS is only one factor that is involved in the induction of intestinal and systemic inflammatory response in patients with IBD.

In fact, in addition to LPS from gut pathogens, additional factors, including dietary components, in particular, sugars, gluten, yeast, and some xenobiotics have been shown to be directly or indirectly responsible for the alteration of the intestinal epithelial tight junction and the induction of intestinal inflammation [31,64,65,66,67,68,69,70,71,72].

In this study, we chose to use LPS because a previous study suggested that LPS at low but clinically relevant concentrations (0.1–1 ng/mL) causes a selective increase in intestinal tight junction proteins by inducing enterocyte membrane expression and localization of TLR4, without causing cell damage or cell death [73]. But using a high pharmacological dose of 50 μg/mL was shown to impair the integrity of the intestinal barrier, leading to rapid cell death in animal models [74].

This is why at high concentrations the inflammatory effect of systemic LPS has been linked to multiple disorders manifesting from gut to brain [15,31,32,33,75,76,77,78,79,80,81,82,83,84]. The increased levels of serum LPS, gut, and BBB antibodies detected in our study, therefore, can explain the increased prevalence of extra-intestinal autoimmune disorders seen in patients with IBD [84]. Even though neurological comorbidities are not frequently reported [10], incidences of psychiatric disorders were shown to be higher in patients with IBD [85,86,87,88].

A breach of the BBB allows for circulating autoantigens to enter the brain and nervous system and thus contribute to neuroautoimmunity. This may explain the moderate to high correlations that were found between IgA and IgM isotype antibodies against LPS, zonulin+occludin, S100B, and AQP4 in both ASCA- and ANCA-positive sera (Table 2 and Table 5). 

The antibodies IgA and IgM in tandem have become the preferred biomarkers for inflammatory gastrointestinal disorders. Due to its polymeric quality, IgA, which is reflective of mucosal immunity, can be transported from the gut to the blood stream, making it a more specific biomarker for the gut. On the other hand, IgM is widely used to identify immune responses in the acute or inflammatory stage, making it an ideal inflammatory biomarker. In fact, our results showed a higher correlation coefficient with the IgA (0.5–0.9) and IgM (0.8–0.9) results. Correlation coefficients with IgG were less statistically significant (0.2–0.6). This shows that IgA and IgM reactivity to LPS puts a patient with IBD at a greater risk for damage to the gut barrier, including disruption of zonulin/occludin, which can result in consequences and manifestations beyond the gastrointestinal tract [89]. 

We also examined the correlation between LPS, gut, and BBB protein antibodies in the control sera. We found a significant correlation between IgA and IgM antibodies against all four tested antigens in about 10% of control subjects who exhibited high levels of antibodies (data not shown). Because we did not have enough clinical information about the control sera, especially the 10% with the elevated antibody levels, we cannot exclude whether or not these individuals had any GI symptomatology or disorders.

The question may be asked, why measure antibodies against LPS, zonulin+occludin, S100B, and AQP4 in blood? Why not directly measure the actual levels of these factors in the blood, instead of the levels of their antibodies?

One answer to this question was given in our earlier research article about the fluctuation of zonulin levels in the blood versus the stability of antibodies [90]. As we discussed in that article, zonulin is an important protein component of tight junction integrity 47,000 Da in size. Certain environmental factors and circumstances can cause it to be released from the lamina propria. It is then presented to the submucosal gut immune system, leading to the generation of an immune response that results in the production of antibodies specifically against it [91].

While the half-life of zonulin in serum has still to be established, it is possible to examine the half-life of other similarly-sized proteins, such as LPS. LPS and zonulin are both involved in the induction of inflammation in type-2 diabetes. The half-life of LPS ranges from 2–4 min in blood [92]. This gives the direct measurement of levels of LPS in the blood a degree of difficulty and uncertainty.

This fluctuation is not unique to proteins such as LPS and zonulin. Take, for instance, the measurement of levels of U1 nuclear antigen in the blood. The detection of elevated blood levels of U1 antigen and circulating autoantibodies against U1 nuclear antigen are held to be the hallmark of systemic lupus erythematosus. However, although U1 antigen antibody levels were demonstrated to be highly stable, the level of U1 antigens fluctuated from day to day. This fluctuation in antigen level in sera may be connected to antibody-antigen complex formation in the circulation [93].

The same caveat applies to measuring the blood level of S100B, which is currently regarded as a reliable biomarker only for acute cases of traumatic brain injury. Using kinetic modeling, researchers demonstrated that S100B concentration changes dramatically over timescales, and that the peak for its level was found to be at 27.2 h after traumatic brain injury [94]. Thus, the predictive value of S100B measurements in blood must be reinforced by accurate timing in sampling [95,96]. 

Similarly, caution should be applied in the utilization as a biomarker of direct levels of aquaporin-4 (AQP4). AQP4 is a water channel protein that conducts water through the cell membrane. It is found in many cells of the human body, including the brain, lung, stomach, and skeletal muscle [97], and is the most prevalent water channel in the central nervous system. AQP4 is believed to be involved in maintaining and regulating the brain’s functions. These same water channel cells are found in plants and bacteria; some studies indicate that homology between human AQP4 and non-human AQP4 and the resulting molecular mimicry may have a role in the development of some neuroautoimmune diseases [98,99]. There is also evidence indicating the involvement of intestinal aquaporin in early stage IBD and in the degrading of the intestinal barrier’s integrity [97,100,101]. 

Additionally, since S100B and AQP4 is expressed not only in the BBB but also in the enteric nervous system, we cannot at this point determine whether the antibodies we detected against S100B and AQP4 originated from the brain or the enteric nerve system. Therefore, S100B or AQP4-mediated inflammatory effects are not limited to the brain, but their over-expression and the immune response mounted against them may also be associated with the onset and maintenance of inflammation in the gut [102,103].

The significant elevation of LPS antibody and its correlation with gut and BBB protein antibodies in ASCA- and ANCA-positive sera but not in healthy controls, as shown in this study, confirms that many patients with IBD indeed suffer from not only from gut inflammation but from systemic inflammation and enhanced gut and BBB permeability to macromolecules [104]. 

It has been posited that intestinal permeability represents an early event that presages the actual onset of IBD. In 2019 Caviglia et al. measured serum as well as fecal zonulin in 118 IBD patients as biomarkers of intestinal permeability [105]. In comparison to controls, serum but not fecal zonulin levels were found to be higher in both CD and UC patients. This elevation of serum level of zonulin and other tight junction proteins indicates that intestinal permeability may be responsible for neurological complications of IBD [23]. At the BBB, S100B and claudin-5 are the most enriched proteins, and their dysfunction has been implicated in neuroinflammatory disorders such as MS, neurodegenerative diseases such as Alzheimer’s, and psychiatric disorders including depression and schizophrenia [56,57,106,107].

Very recently, Michael Maes, in collaboration with our laboratory, reported increased plasma level of antibodies against Gram-negative bacteria, paracellular tight and adherens junction, transcellular cytoskeletal proteins, gut vascular barrier and blood brain barrier proteins in patients with deficit schizophrenia. Maes concluded that bacterial translocation and production of bacterial toxins is responsible for the upregulation and breakdown of tight and adherens junction in the gut, vascular barrier, and blood brain barrier in patients with schizophrenia [58,59,60].

We agree with Maes’s findings, and we therefore believe that gut dysbiosis, the release of LPS, and the production of LPS antibodies against them in a subgroup of patients with Crohn’s disease and ulcerative colitis should be detected and addressed at the earliest stage possible. Otherwise, the consequences of this breakdown in the barriers by bacterial toxins could be neuroinflammation, neuroautoimmunity, neurodegeneration, and neuropsychiatric disorders.

There are several limitations to our study:

1. We did not have any clinical information about our so-called healthy controls other than that they were screened based on a questionnaire and were negative for HIV and hepatitis-C antibodies. The additional information is important for the analysis of data from about 10% of controls who produced significant levels of antibodies against LPS as well as the barrier proteins.
2. Likewise, other than their positivity or negativity when screened for ASCA or ANCA, we also did not have additional information about the clinical symptomatology of our ASCA- and ANCA-positive samples.
3. We do not know whether the antibodies that we detected against AQP4 and S100B were made against these molecules in the brain, in the GI tract, or against both the gut and the brain glia and astrocytes.
4. Due to our sample size and optical density variances, the confidence intervals were wide. This is due to the fact that only one-third of the ASCA- and ANCA-positive sera were highly reactive. However, there were still significant increased odds of 6- to 40-fold with the lowest tail of the confidence intervals.

While we agree that these limitations should be addressed in future studies, our present findings suggest that individuals diagnosed with Crohn’s disease and ulcerative colitis with ASCA- and ANCA-positive antibodies have increased odds and correlations for both intestinal permeability and BBB permeability when compared to healthy subjects (ASCA- and ANCA-negative).

## 4. Materials and Methods 

### 4.1. Blood Samples and Antigens

Commercially available sera of 24 patients with Crohn’s disease and 24 sera from patients with celiac disease were purchased from The Binding Site (San Diego, CA, USA), Inova (San Diego, CA, USA), Trina International (Nanikon, Switzerland), Diamedix (Hialeah, FL, USA), and Innovative Research (Novi, MI, USA). We also purchased from Innovative Research (Novi, MI, USA) additional blood samples obtained from donors who had been screened for anti-*Saccharomyces cerevisiae* antibodies (ASCA) and anti-neutrophil cytoplasmic antibodies (ANCA) positivity or negativity. In this way we accumulated a total of 94 samples that were positive for ASCA and 94 samples that were positive for ANCA. We tested these samples using a kit procured from Inova, and compared them with sera from 188 healthy controls. Sera were assessed for serum immunoglobulin IgG, IgA, and IgM reactivities to LPS, as well as zonulin+occludin, human aquaporin (AQP4), and S100B. LPS from *E. coli*, *Salmonella*, *Shigella*, *Klebsiella*, and *Pseudomonas* were purchased from Sigma-Aldrich^®^ (St. Louis, MO, USA). Occludin, zonulin, S100B and aquaporin-4 were purchased from Bio-Synthesis^®^ (Lewisville, TX, USA). 

### 4.2. Antibody Measurement

Antibodies against these proteins were measured using enzyme-linked immunosorbent assay (ELISA) as previously described [48].

LPS, zonulin+occludin, S100B, and human AQP4 at a concentration of 1 mg/mL were dissolved in 0.01 M Tris buffer and diluted 1:50 in 0.1M carbonate buffer at pH 9.5. 100 μL of each diluted antigen was added to each well of the microtiter plate. Plates were incubated for 24 h at 4 °C and then washed three times with 200 μL 0.01 M PBS containing 0.05% Tween 20 at a pH of 7.4. After washing, 200 μL of 2% bovine serum albumin was added to each well to prevent non-specific binding of the antibody to the plate.

Plates were washed, and then 100 μL of serum diluted 1:100 in serum diluent were added to duplicate wells coated with each antigen. Plates were incubated for an additional 1 h at room temperature. The plates were then washed five times with Tris-buffered saline (TBS)-Tween. Alkaline phosphatase-labeled anti-human IgG, IgM, or IgA antibodies were then added to all wells and incubated again for 1 h at room temperature. The enzyme reaction was started by adding 100 μL of substrate at a concentration of 1 mg/mL. The reaction was stopped by the addition of 50 μL of 1 N NaOH, and the samples were read by an ELISA reader; the optical densities were recorded. In addition to blank wells, several wells were coated with non-specific proteins such as human serum albumin (HSA) and rabbit serum. All reagents were added, and their ODs were recorded and used as controls for detecting the background and non-specific reaction.

### 4.3. Statistical Analysis

Statistical analysis was performed using GraphPad Prism 6.0 software (San Diego, CA, USA). The diagnostic values of the indirect ELISA assays was evaluated by the receiver operating characteristic (ROC) curve. The optimal cutoff values were chosen according to ROC analysis, setting specificity at 0.31 since only about one third of the patients reacted strongly to LPS and barrier proteins. Additional data analysis was performed using STATA 14.2 software. Logistic regression, t-tests, and Pearson’s correlation coefficients were used to analyze the data. Bonferroni adjustments were performed to avoid a false discovery rate for multiple comparisons.

### 4.4. Research Ethics

This study was conducted in accordance with the Declaration of Helsinki, and the research received IRB approval from Partner’s Human Research Committee at Massachusetts General Hospital (Protocol #2106P002738/ MGH: date of approval 20 December 2016). 

## 5. Conclusions

Because bacterial toxins such as LPS produced by Gram-negative bacteria can damage the gut and blood-brain barriers, which are the two major gateways to autoimmune, neuroinflammatory, neurodegenerative, and neuropsychiatric disorders, we conclude that patients with IBD should be screened for LPS IgG, IgM, and IgA antibodies and their correlation with barrier protein antibodies in an effort to prevent BBB damage and its neurologic comorbidities. By regulating the levels of BBB and intestinal tight junction proteins, it is possible to abrogate disease symptoms, not only in Crohn’s disease and ulcerative colitis, but in associated disorders as well.

However, much more remains to be done regarding understanding the major factors that are involved in the pathogenesis of IBD in order to successfully implement lifestyle modifications.

## Figures and Tables

**Table 1:** Odds ratios for developing barrier antibodies with anti-*Saccharomyces cerevisiae* antibodies (ASCA) positive subjects compared with ASCA-negative subjects.

Barrier Protein Antibodies	Odds Ratio	Confidence Interval	*p*-Value
S100B IgA	36	11–110	<0.0001
S100B IgG	65	21–191	<0.0001
S100B IgM	5	3–10	<0.0001
Aquaporin-4 IgA	149	40–533	<0.0001
Aquaporin-4 IgG	96	30–305	<0.0001
Aquaporin-4 IgM	9	4–20	<0.0001
Zonulin+Occludin IgA	20	8–56	<0.0001
Zonulin+Occludin IgG	13	6–29	<0.0001
Zonulin+Occludin IgM	8	4–17	<0.0001
Lipopolysaccharides IgA	107	34–340	<0.0001
Lipopolysaccharides IgG	8	4–16	<0.0001
Lipopolysaccharides IgM	11	5–24	<0.0001

**Table 2:** Correlation coefficients and *p*-values for barrier protein antibodies for anti-*Saccharomyces cerevisiae* antibodies (ASCA) positive subjects.

Correlations	r-Value	*p*-Value
Zonulin+Occludin IgA and S100B IgA	0.8	<0.0001
Zonulin+Occludin IgG and S100B IgG	0.3	<0.0001
Zonulin+Occludin IgM and S100B IgM	0.8	<0.0001
Lipopolysaccharide IgA and S100B IgA	0.7	<0.0001
Lipopolysaccharide IgG and S100B IgG	0.2	<0.0001
Lipopolysaccharide IgM and S100B IgM	0.8	<0.0001
Zonulin+Occludin IgA and Aquaporin-4 IgA	0.6	<0.0001
Zonulin+Occludin IgG and Aquaporin-4 IgG	0.5	<0.0001
Zonulin+Occludin IgM and Aquaporin-4 IgM	0.8	<0.0001
Lipopolysaccharide IgA and Aquaporin-4 IgA	0.5	<0.0001
Lipopolysaccharide IgG and Aquaporin-4 IgG	0.3	<0.0001
Lipopolysaccharide IgM and Aquaporin-4 IgM	0.8	<0.0001
Aquaporin-4 IgA and S100B IgA	0.7	<0.0001
Aquaporin-4 IgG and S100B IgG	0.6	<0.0001
Aquaporin-4 IgM and S100B IgM	0.9	<0.0001
Lipopolysaccharide IgA and Zonulin+Occludin IgA	0.5	<0.0001
Lipopolysaccharide IgG and Zonulin+Occludin IgG	0.5	<0.0001
Lipopolysaccharide IgM and Zonulin+Occludin IgM	0.7	<0.0001

**Table 3:** Numbers and % elevation of IgG, IgM, and IgA antibodies against lipopolysaccharides (LPS), zonulin+occludin, S100B and aquaporin-4 (AQP4) in sera positive or negative for ASCA or ANCA.

Antigens		ASCA- (Controls)		ASCA+		ANCA- (Controls)		ANCA+	
LPS	IgG	11/94	12%	29/94	31%	2/94	2%	28/94	30%
	IgM	8/94	9%	31/94	33%	6/94	6%	29/94	31%
	IgA	10/94	11%	28/94	30%	4/94	4%	29/94	31%
Zonulin + Occludin	IgG	12/94	13%	28/94	30%	0/94	0%	28/94	30%
	IgM	8/94	9%	28/94	30%	5/94	5%	29/94	31%
	IgA	11/94	12%	28/94	30%	6/94	6%	29/94	31%
S100B	IgG	5/94	5%	27/94	29%	6/94	6%	29/94	31%
	IgM	11/94	12%	28/94	30%	8/94	9%	29/94	31%
	IgA	2/94	2%	29/94	31%	5/94	5%	30/94	32%
AQP4	IgG	5/94	5%	28/94	30%	3/94	3%	29/94	31%
	IgM	7/94	7%	28/94	30%	7/94	7%	28/94	30%
	IgA	4/94	4%	29/94	31%	3/94	3%	30/94	32%

**Table 4:** Odds ratios for developing barrier antibodies with anti-neutrophil cytoplasmic antibodies (ANCA) positive subjects compared with the odds of developing barrier protein antibodies with controls.

Barrier Protein Antibodies	Odds Ratio	Confidence Interval	*p*-Value
S100B IgA	47	16–134	<0.0001
S100B IgG	27	9–86	<0.0001
S100B IgM	9	4–20	<0.0001
Aquaporin-4 IgA	87	27–281	<0.0001
Aquaporin-4I gG	95	27–326	<0.0001
Aquaporin-4 IgM	9	4–20	<0.0001
Zonulin+Occludin IgA	11	5–26	<0.0001
Zonulin+Occludin IgG	16	7–42	<0.0001
Zonulin+Occludin IgM	8	4–16	<0.0001
Lipopolysaccharides IgA	9	4–19	<0.0001
Lipopolysaccharides IgG	38	15–98	<0.0001
Lipopolysaccharides IgM	13	6–30	<0.0001

**Table 5:** Correlation coefficients and *p*-values for barrier protein antibodies for anti-neutrophil cytoplasmic antibodies (ANCA) positive subjects.

Correlations	r-Value	*p*-Value
Zonulin+Occludin IgA and S100B IgA	0.6	<0.0001
Zonulin+Occludin IgG and S100B IgG	0.6	<0.0001
Zonulin+Occludin IgM and S100B IgM	0.7	<0.0001
Lipopolysaccharide IgA and S100B IgA	0.6	<0.0001
Lipopolysaccharide IgG and S100B IgG	0.4	<0.0001
Lipopolysaccharide IgM and S100B IgM	0.4	<0.0001
Zonulin+Occludin IgA and Aquaporin-4 IgA	0.7	<0.0001
Zonulin+Occludin IgG and Aquaporin-4 IgG	0.6	<0.0001
Zonulin+Occludin IgM and Aquaporin-4 IgM	0.8	<0.0001
Lipopolysaccharide IgA and Aquaporin-4 IgA	0.7	<0.0001
Lipopolysaccharide IgG and Aquaporin-4 IgG	0.5	<0.0001
Lipopolysaccharide IgM and Aquaporin-4 IgM	0.5	<0.0001
Aquaporin-4 IgA and S100B IgA	0.8	<0.0001
Aquaporin-4 IgG and S100B IgG	0.7	<0.0001
Aquaporin-4 IgM and S100B IgM	0.9	<0.0001
Lipopolysaccharide IgA and Zonulin+Occludin IgA	0.6	<0.0001
Lipopolysaccharide IgG and Zonulin+Occludin IgG	0.3	0.001
Lipopolysaccharide IgM and Zonulin+Occludin IgM	0.4	<0.0001
